# Prehospital cooling in cardiac arrest - the next frontier?

**DOI:** 10.1186/1757-7241-17-54

**Published:** 2009-10-12

**Authors:** Eldar Søreide

**Affiliations:** 1 Department of Anaesthesia and Intensive Care, Stavanger University Hospital, Stavanger, Norway

## Abstract

Therapeutic hypothermia (TH) in unconscious survivors of out-of-hospital cardiac arrest (OHCA) is now a well-documented part of post-resuscitation care. Implementation of TH into daily clinical practice has been far more successful in the Scandinavian countries than in the rest of the world. Still, many questions remain. One of them is whether prehospital cooling will result in better outcomes.

Therapeutic hypothermia (TH) in unconscious survivors of out-of-hospital cardiac arrest (OHCA) is now a well-documented part of post-resuscitation care [1,2]. Implementation of TH into daily clinical practice has been far more successful in the Scandinavian countries than in the rest of the world [3,4]. Still, many questions remain unanswered:

• Is there a better, safer and more rapid way of cooling these patients?

• Does rapid cooling necessarily mean prehospital cooling?

• And, will rapid prehospital cooling translate into higher survival rates and better neurological outcomes?

In this issue of the Journal, two international research groups within this exciting and rapidly progressing field of critical care medicine have reviewed the present knowledge on prehospital cooling in OHCA [5,6]. Behringer et al [5] give an excellent overview on what is known about prehospital preservative and resuscitative hypothermia. Their main focus is on resuscitative hypothermia - meaning cooling initiated after return of spontaneous circulation (ROSC). Both non-invasive cooling pads and IV. infusion of ice-cold fluids have been shown to be feasible alternatives in the prehospital environment, securing earlier induction of the cooling process. What is lacking is convincing human data on improved clinical outcomes. Kämäräinen et al [6] come to the same conclusion. In their review they also mentioned a specially designed cooling cap as a possible method of (selective) brain cooling. They also review the present human data on prehospital intra-arrest cooling. After much promising animal data, little more than feasibility and safety data has been published in humans. However, this may all change in the next months to come.

The Australian trial on prehospital cooling versus in-hospital cooling in OHCA survivors (RICH-trial) has been presented at an international medical meeting. The trial now has been broadened to include intra-arrest cooling as well (Stephen Bernard, personal communication). The recent 3^rd ^International Hypothermia Symposium  in Lund, Sweden also presented break-through research in the field, one being intra-arrest trans-nasal cooling with a highly evaporative perfluorocarbon spray. The technique has been shown to improve ROSC rates and secure very rapid brain cooling in animal studies [7]. Preliminary Swedish results from a multi-centre European trial indicate that intra-arrest trans-nasal cooling using this commercial available device may improve ROSC rates and survival also in humans. However, the promising results have yet to be published in peer-reviewed journals. We therefore need to be cautious before jumping to conclusions affecting our clinical practice.

One concern raised in the current reviews [5,6] is the lack of on-going hospital cooling in patients brought to hospital after prehospital cooling had been commenced. Some studies actually reported active hospital warming of patients cooled during ambulance transport. This is probably worse than no cooling at all. This should act as a reminder to us all that for the Chain of Survival (Figure 1) to get stronger, clinicians inside and outside hospitals must work together. Together, we should decide not only how and when to cool, but also who to cool. It does not make sense anymore to limit cooling to VF cardiac arrests only [1,2,8]. We cool the brain because it suffers from a combination of anoxic and re-perfusion injury, not because of a specific heart rhythm. If you decide to treat unconscious survivors of OHCA actively in the ICU, TH should be part of standard care. Whether you should move the cooling into ambulances or the homes of cardiac arrest victims is another discussion not yet settled. In order to decide what your future treatment strategy should be, a very good starting point is to read the two present reviews on prehospital cooling [5,6].

**Figure 1:**
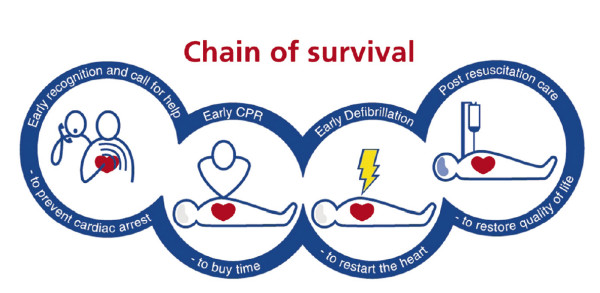
**The ERC 2005 Chain of Survival**. From: Jerry Nolan, Jasmeet Soar, and Harald Eikeland. Resuscitation 2006, 71, 270--271
